# Prognostic role of the Glasgow prognostic score and modified Glasgow prognostic score in patients with renal cell carcinoma undergoing immunotherapy: a meta-analysis

**DOI:** 10.1016/j.clinsp.2026.101008

**Published:** 2026-07-17

**Authors:** Xiangning Zhang, Xiaoye Li, Yajun Xie, Ning Shi

**Affiliations:** Department of Pharmacy, The Ninth Medical Center, Chinese PLA General Hospital, Beijing, China

**Keywords:** Renal cell carcinoma, Immune checkpoint inhibitors, Glasgow prognostic score, GPS, mGPS, Meta-analysis

## Abstract

**Background:**

Overall Survival (OS) in patients with urological cancers can be predicted by the Glasgow Prognostic Score (GPS) and modified GPS (mGPS). However, the predictive value of the GPS and mGPS in individuals with Renal Cell Carcinoma (RCC) receiving immunotherapy remains inconclusive. The present analysis examined how the GPS and mGPS predict prognosis in RCC patients treated with immunotherapy.

**Methods:**

Studies available before March 18, 2025, were systematically searched in PubMed, Web of Science, Embase, and Cochrane. To determine prognostic effects in RCC, Hazard Ratios (HRs) and 95 % Confidence Intervals (95 % CIs) were estimated.

**Results:**

This meta-analysis encompassed nine eligible studies. Patients with high GPS/mGPS (1/2/1‒2) had a significantly higher risk of death compared with those with low GPS/mGPS (0): OS (HR = 2.48; 95 % CI 2.05–2.99; *p* < 0.001) and Progression-Free Survival (PFS) (HR = 1.84; 95 % CI 1.61–2.11; *p* < 0.001). Subgroup analysis revealed that high-GPS/mGPS RCC patients in Asia (HR = 3.19; 95 % CI 1.63–6.23) and Europe (HR = 2.90; 95 % CI 2.25–3.74) had higher mortality risk compared with those in North America (HR = 1.88; 95 % CI 1.38–2.54). Furthermore, the prognostic impact of GPS/mGPS was more evident in metastatic patients (HR = 3.18; 95 % CI 2.43–4.14; *p* < 0.001) than in those without metastasis (HR = 1.94; 95 % CI 1.49–2.52; *p* < 0.001). mGPS-only analysis confirmed poor prognosis (HR = 2.56; 95 % CI 1.95–3.38; *p* < 0.001); metastatic burden drove heterogeneity (meta-regression *p* < 0.05).

**Conclusion:**

GPS/mGPS may serve as effective biomarkers for predicting survival in RCC patients receiving ICI therapy. High GPS and mGPS are associated with increased mortality risk. Further randomized controlled trials are warranted to explore the prognostic potential of GPS/mGPS.

## Highlights

• GPS predicts poor OS and PFS in RCC patients treated with ICIs.
• GPS shows region-specific prognostic value in RCC patients receiving ICIs.
• GPS retains prognostic value mainly in metastatic RCC populations.
• GPS may support pre-immunotherapy risk stratification in RCC.

## Introduction

Renal Cell Carcinoma (RCC) constitutes around 85 % of primary renal cancers, emerging as the leading urinary malignancy and placing considerable strain on human health.^1^ Globally, in 2022, about 434,840 new cases were reported, reflecting a rising incidence of RCC.^2^ Localized RCC is predominantly managed by surgical removal. Although multimodal therapy centered on surgery has improved survival outcomes, prognosis in advanced disease remains unsatisfactory owing to recurrence and metastasis. Approximately half of locally advanced patients who experience postoperative recurrence require systemic therapy. In the absence of effective prognostic biomarkers, disease management becomes challenging. Consequently, identifying reliable and accurate prognostic biomarkers has become increasingly important.

Immune Checkpoint Inhibitors (ICIs) were first approved for advanced renal cancer in 2015 and became a standard therapeutic strategy in 2018.^3^ Although ICI therapy has improved RCC prognosis, predictive biomarkers commonly used in other cancers, such as PD-L1 expression and Tumor Mutational Burden (TMB), demonstrate limited efficacy in predicting ICI response in RCC and fail to effectively identify potential responders, thereby substantially increasing the disease burden.^4^^,^^5^ Furthermore, patient responses to ICIs vary considerably.^6^ Therefore, identifying prognostic factors is critical for predicting which patient populations are more likely to achieve favorable responses, and such factors may play a pivotal role in clinical decision-making.

Systemic inflammatory response has been identified as a contributing factor to ICI resistance across multiple malignancies, including RCC. Systemic inflammation further facilitates immune evasion in RCC by shaping an immunosuppressive tumor microenvironment. As described by Gutic et al., the PD-1/PD-L1 axis serves as a central regulator of T-cell activation, whereas the systemic inflammatory state reflected by GPS (characterized by elevated CRP and hypoalbuminemia) creates an immunosuppressive microenvironment enriched with IL-6, VEGF, and Myeloid-Derived Suppressor Cells (MDSCs), which impairs CD8⁺ T-cell infiltration and function. Consequently, PD-1/PD-L1 blockade by ICIs may fail to restore antitumor immunity, providing a biological rationale for GPS as a predictor of ICI treatment failure.^7^ The Glasgow Prognostic Score (GPS), a marker of systemic inflammation, is determined by measurements of serum CRP and albumin. Albumin serves as a biomarker of nutritional status, while CRP is a sensitive indicator of inflammatory response and has been demonstrated to promote the formation of immunosuppressive tumor microenvironments and tumor cell growth.^8^ A GPS score of 2 is assigned when elevated CRP (> 10 mg/L) coexists with hypoalbuminemia (albumin < 35 g/L), a score of 1 is assigned when only one parameter is abnormal, and a score of 0 is assigned when both parameters are within normal ranges. In contrast, the mGPS emphasizes that hypoalbuminemia contributes to the score only when CRP is elevated; if CRP ≤10 mg/L, mGPS is 0 regardless of albumin level, thereby placing greater weight on the inflammatory component of the score.^9^^,^^10^ The mGPS demonstrated that fewer cases with hypoalbuminemia had elevated CRP levels, suggesting that hypoalbuminemia alone may not be associated with poor survival.^11^ Studies have indicated that the mGPS system is more effective in reflecting systemic inflammatory response than individual inflammatory markers alone.^12^ Across different malignancies, including RCC,^13^, ^14^, ^15^ elevated GPS/mGPS is consistently correlated with adverse prognosis, confirming its strong predictive value for survival.^15^^,^^16^ In the context of ICI therapy, GPS and mGPS have been used as prognostic indicators for patient outcomes.^17^ However, evidence to date shows inconsistency concerning the prognostic importance of GPS/mGPS in RCC patients.^18^, ^19^, ^20^, ^21^, ^22^ Therefore, this meta-analysis integrates available findings to clarify the relationship between GPS/mGPS and prognosis in RCC patients receiving ICIs, aiming to facilitate clinical prediction of disease progression during RCC treatment.

## Materials and methods

The methodology adhered to the PRISMA (Preferred Reporting Items for Systematic Reviews and Meta-Analyses) standards.^23^ Registration of the protocol was completed with PROSPERO (International Prospective Register of Systematic Reviews, CRD420251019152).

### Search strategy

Searches of PubMed, Embase, the Cochrane Library, and Web of Science identified all publications up to March 18, 2025. The language was restricted to English. Search terms comprised both Medical Subject Headings (MeSH) and free-text entries such as “Glasgow Prognostic Score”, “immunotherapy”, “immune checkpoint inhibitors”, and “renal cell carcinoma”. Reference lists and grey literature were manually screened to supplement the database search. The specific search approach is available in Supplementary Material 1.

### Inclusion and exclusion criteria

Eligibility criteria included: 1) RCC patients receiving ICIs; 2) Exposure group: GPS/mGPS scores of 1 or 2; comparator: GPS/mGPS score of 0; 3) Cohort study design; 4) Outcomes: adjusted OS and PFS.

Exclusion criteria included: 1) Non-human studies, case reports, non-original literature, and conference abstracts; 2) Studies with poor-quality or incomplete data; 3) Duplicate publications; 4) Studies without full-text access; 5) Studies with overlapping patient populations.

### Data extraction

Retrieved records were imported into EndNote. Initial screening of titles and abstracts was independently performed by two reviewers, with eligible records undergoing full-text assessment in the second phase. Any disagreements during screening were addressed through consensus or adjudicated by an additional evaluator (Yajun Xie). Two independent researchers extracted the data simultaneously, employing a structured digital template. Extracted information included the first author, publication year, randomization and blinding, treatment regimen, region, sample size, sex, age, and outcomes.

### Quality assessment

Study quality was independently assessed by two investigators (Xiangning Zhang and Xiaoye Li) with the Newcastle-Ottawa Scale (NOS), incorporating eight items covering selection, comparability, and exposure.^24^ Studies were graded on a 0–9 scale under the NOS system, with a threshold of 6 differentiating high- and low-quality studies.

### Statistical analysis

STATA version 15.1 was employed to conduct all statistical computations. To explore the link between GPS/mGPS and prognosis among RCC patients under ICI therapy, HRs with 95 % CIs were computed. Given the objective heterogeneity among the included studies, the authors employed a random-effects model for all meta-analysis results. For outcomes exhibiting significant heterogeneity, the authors performed meta-regression to explore potential sources of between-study variation. To ensure dependability, sensitivity analyses were performed to examine whether the findings remained consistent and reliable. To detect heterogeneity origins, subgroup analyses were stratified by geography, metastasis presence, therapeutic protocol, and cohort size. Funnel plots and Egger’s test were used to detect publication bias, with statistical significance defined as *p* < 0.05.

## Results

### Study selection

**Fig. 1:**
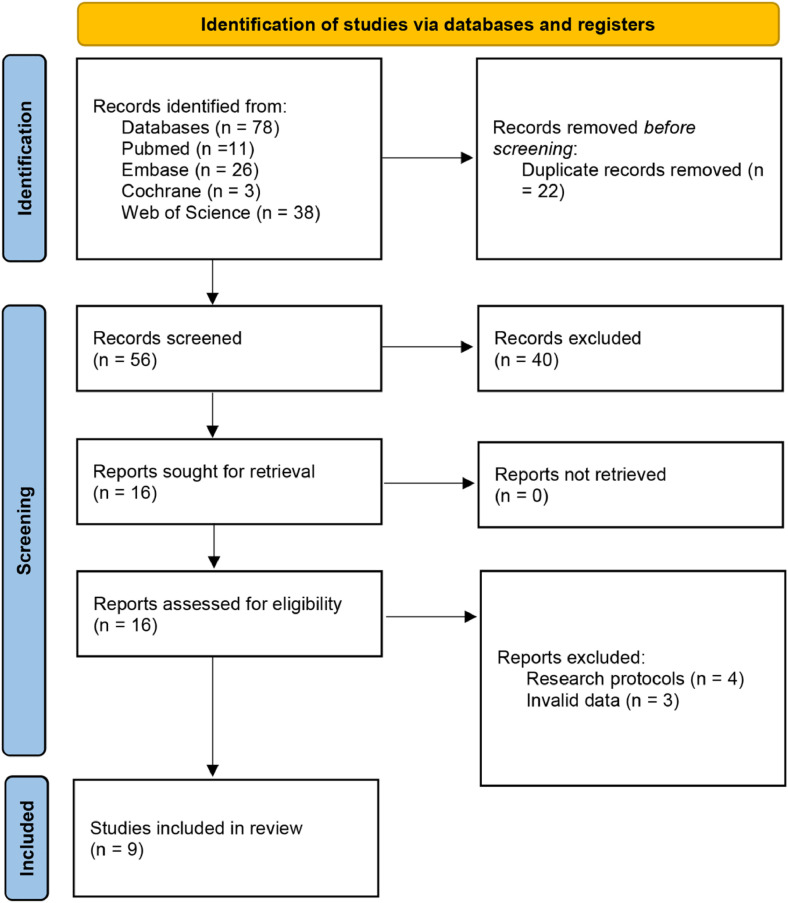
Flow diagram for the selection of studies and specific reasons for exclusion from the present meta-analysis.

Of the 78 records initially identified, 22 duplicates were removed. Following title and abstract review, 40 studies were removed, while the remainder were assessed for full-text eligibility. A final selection of 9 studies was retained for analysis (Supplementary Material 2). The selection process is illustrated in Fig. 1.

### Baseline characteristics and quality assessment

The 9 included studies were conducted across three regions (North America, Asia, and Europe), involving a total of 1747 patients. Of these, 71.09 % were male, and 28.91 % were female, with a mean age ranging from 59.48- to 66.68-years. The baseline characteristics of the selected studies are summarized in Table 1. All included studies achieved Newcastle-Ottawa Scale scores above 6, indicating high methodological quality (Supplementary Material 3).

**Table 1:** Details of included RCTs and characteristics of the included patients.

Author	Year	Study design	Combination	Region	Sample size	Gender (Male/ Female)	Age	Metastatic condition	Outcome	Categories
Brown, J. T.	2021	Retrospective Cohort study	Combination therapy	America	156	108/48	63.4 (12.63)	Metastasis	OS, PFS	mGPS
Fujiwara, R.	2020	Retrospective Cohort study	Monotherapy	Asia	45	NA	62 (3.18)	Metastasis	OS	mGPS
Minichsdorfer, C.	2022	Retrospective Cohort study	Combination therapy	Europe	114	74/40	59.48 (12.97)	Non-metastasis	OS	GPS
Saal, J.	2025	Retrospective Cohort study	Combination therapy	Europe	121	85/36	NA	Non-metastasis	OS, PFS	mGPS
Sato, M. T.	2022	Retrospective Cohort study	Monotherapy	Asia	102	83/19	65.89 (11.97)	Metastasis	OS, PFS	mGPS
Walach, M. T.	2025	Retrospective Cohort study	Combination therapy	Europe	53	44/9	63.48 (3.35)	Metastasis	OS	mGPS
Yildirim, A.	2025	Retrospective Cohort study	Combination therapy	America	401	283/118	65.59 (13.03)	Non-metastasis	OS, PFS	mGPS
Noguchi, Go	2020	Retrospective Cohort study	Monotherapy	Asia	64	51/13	66.68 (12.44)	Metastasis	PFS	GPS
Saal, Jonas	2023	Retrospective Cohort study	Combination therapy	Europe	691	514/177	NA	Metastasis	OS, PFS	mGPS

NA, Not Available.

### Meta-analysis results

#### Overall survival (OS)

**Fig. 2:**
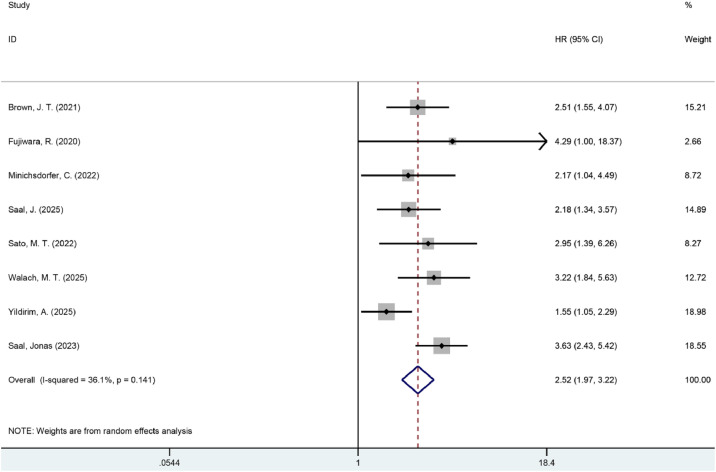
Forest plots of the HRs and 95 % CIs for OS by GPS in Renal cell carcinoma treated with immune checkpoint inhibitors.

Eight studies involving 1683 patients reported OS data. The analysis proceeded using a random-effects model. As shown in Fig. 2, patients with high GPS/mGPS demonstrated significantly higher mortality risk compared with those with low GPS/mGPS (HR = 2.48; 95 % CI 2.05–2.99; *p* < 0.001).

#### Subgroup analyses

**Fig. 3:**
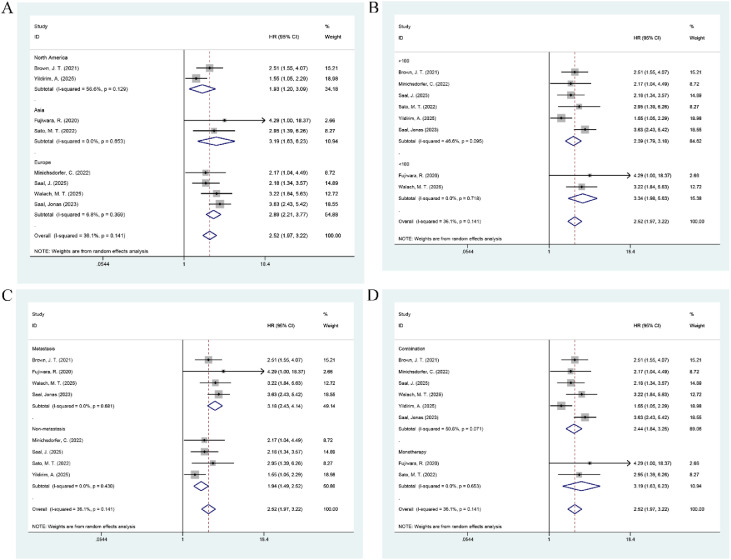
Forest plot of subgroup analysis of the association between Glasgow Prognostic Score (GPS) and Overall Survival (OS) in patients with Renal cell carcinoma. Region (A), sample size (B), metastatic condition (C), and therapeutic combinations (D).

Analyses stratified by region, sample size, metastatic condition, and therapeutic combinations were carried out as subgroup evaluations (Fig. 3A–D). Regional subgroup analysis revealed that high-GPS/mGPS RCC patients in Asia (HR = 3.19; 95 % CI 1.63–6.23; *p* < 0.001) and Europe (HR = 2.90; 95 % CI 2.25–3.74; *p* < 0.001) had higher mortality risk than those in North America (HR = 1.88; 95 % CI 1.38–2.54; *p* < 0.001). Subgroup analysis by metastatic status identified metastasis as a potential source of heterogeneity (*I*^2^ = 36.1 %; *p* = 0.010). Compared with non-metastatic subjects, GPS/mGPS had a stronger link to mortality among metastatic patients (HR = 3.18; 95 % CI 2.43–4.14; *p* < 0.001, vs. HR = 1.94; 95 % CI 1.49–2.52; *p* < 0.001), test of group difference, *p* = 0.01. The test of group difference for the other subgroup analyses (region, combination therapy, and sample size) was 0.30, 0.47, and 0.27, respectively. Subgroup analyses by combination therapy and sample size revealed no significant differences or sources of heterogeneity.

#### The association between mGPS and overall survival (OS)

**Fig. 4:**
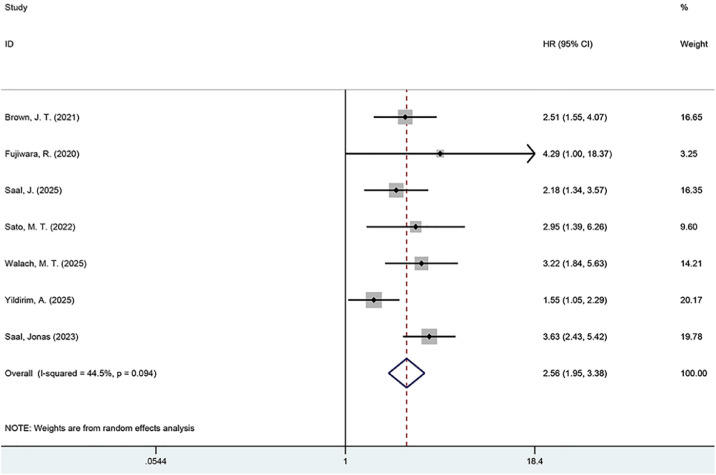
Forest plots of the HRs and 95 % CIs for OS by mGPS in Renal cell carcinoma treated with immune checkpoint inhibitors.

Seven studies involving 1569 patients examined the association between mGPS and OS. As shown in Fig. 4, elevated mGPS was significantly associated with increased mortality risk in RCC patients (HR = 2.56; 95 % CI 1.95–3.38; *p* < 0.001).

#### Meta-regression

Meta-regression analyses were conducted for sample size and metastatic status to explore their potential influence on pooled results. Metastatic status (*p* < 0.05) showed statistically significant regression results, suggesting that metastatic status may be a potential source of heterogeneity for OS outcomes. Sample size (*p* = 0.79) did not demonstrate statistical significance (*p* > 0.05).

#### Progression-free survival (PFS)

**Fig. 5:**
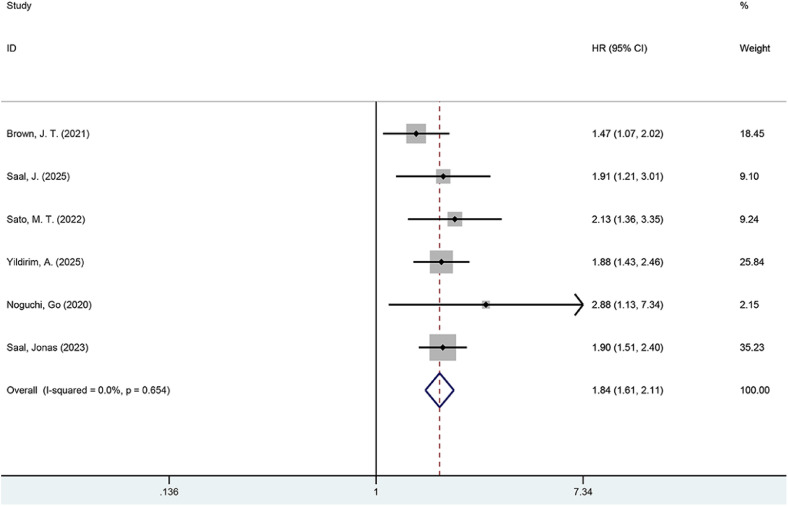
Forest plots of the HRs and 95 % CIs for PFS by GPS/mGPS in renal cell carcinoma treated with immune checkpoint inhibitors.

Six studies involving 1174 patients reported PFS data. Given *I*^2^ = 0 % and *p* = 0.654, the fixed-effects model was adopted. Fig. 5 demonstrates that elevated GPS/mGPS showed a strong association with greater progression risk (HR = 1.84; 95 % CI 1.61–2.11; *p* < 0.001).

### Sensitivity analysis

To evaluate the robustness of the meta-analysis findings, the authors conducted a sensitivity analysis using a random-effects model. The exclusion of single studies had a negligible impact on pooled estimates, affirming the consistency of findings.

### Publication bias

**Fig. 6:**
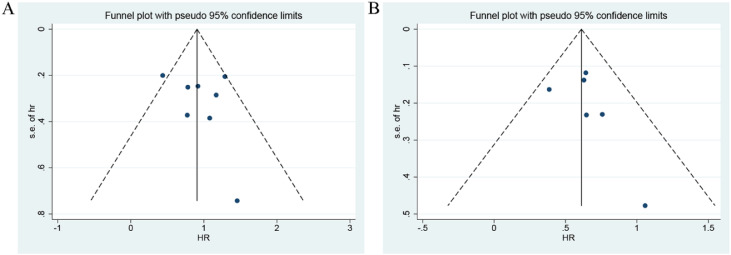
Funnel plots of GPS/mGPS for (A) overall survival and (B) progression-free survival.

Potential publication bias was assessed through funnel plots and Egger’s test. Funnel plots for both OS and PFS showed symmetrical distribution (Fig. 6A and B). Egger's test revealed no evidence of publication bias for OS (*p* = 0.507) or PFS (*p* = 0.371), as both exceeded 0.05.

## Discussion

The study synthesized available data to assess the prognostic utility of GPS/mGPS in RCC patients managed with ICIs. Nine studies were included, demonstrating that GPS/mGPS is an important prognostic factor in this patient population. High GPS/mGPS was closely associated with poorer OS and PFS. Subgroup analyses revealed that high-GPS/mGPS RCC patients from Asia and Europe had higher mortality rates than those from North America, and GPS/mGPS showed a stronger prognostic association in patients with metastatic disease. Notably, among the included studies, only one employed the standard GPS, while the remainder used mGPS. Metastatic status may partly account for the observed heterogeneity in overall survival; however, this finding should be interpreted with caution, as it is derived from study-level rather than patient-level data and warrants confirmation through individual patient-data meta-analyses.

As a composite inflammation-nutrition index integrating serum C-Reactive Protein (CRP) and albumin levels, GPS/mGPS requires only routine blood tests for assessment. Owing to its simplicity, cost-effectiveness, and high reproducibility, it has emerged as a widely investigated potential prognostic tool.^14^^,^^16^ To enhance prognostic accuracy across various cancer types, McMillan et al. refined GPS into mGPS, which diminishes the role of albumin while highlighting the importance of CRP.^11^ For GPS, a score of 0 is determined by both CRP and albumin levels; for mGPS, a score of 0 is determined solely by CRP, irrespective of albumin level. For mGPS of 1, even if albumin is normal, elevated CRP alone yields a score of 1. For a score of 2, GPS and mGPS share identical criteria.^11^ GPS/mGPS has demonstrated prognostic value across multiple cancer types and treatment stages.^25^, ^26^, ^27^ The present findings suggest that GPS/mGPS can serve as a prognostic predictor for RCC patients receiving ICI therapy, with the core conclusions predominantly derived from studies employing the mGPS scoring system.

Multiple risk stratification systems exist for metastatic RCC (mRCC) prognostication. The International Metastatic RCC Database Consortium (IMDC) risk score represents the most widely used prognostic stratification tool for mRCC. This model incorporates six baseline parameters: time from diagnosis to systemic therapy, Karnofsky Performance Status (KPS), corrected calcium, and complete blood count parameters, including hemoglobin, neutrophil count, and platelet count. Each item scores 1 point, yielding a total score of 0–6: 0 indicates favorable risk, 1–2 indicates intermediate risk, and ≥ 3 indicates poor risk, corresponding to median overall survival of approximately 40–50 months, 20–25 months, and < 12-months in the contemporary immunotherapy era, respectively. However, the IMDC criteria were developed based on survival data from patients receiving anti-angiogenic therapies (including sunitinib, sorafenib, and bevacizumab) during the targeted therapy era, and their application is relatively cumbersome, with some subjective elements such as performance status assessment. Brown et al. demonstrated in an RCC cohort study that mGPS possessed prognostic value in patients receiving any first-line immunotherapy, with predictive capability for survival outcomes comparable to the IMDC risk score.^15^ Fujiwara et al. reported that incorporating mGPS into the IMDC model improved the Concordance index (C-index), suggesting that a composite “IMDC + mGPS” score merits prospective evaluation.^18^

The association between malignant progression of RCC and systemic inflammation is particularly well-established. Substantial evidence indicates that systemic inflammation portends poor response to ICIs in genitourinary cancers, including RCC.^28^^,^^29^ Systemic inflammation is associated with nutritional and functional deterioration in advanced cancer patients, with consequent poor prognosis and quality of life indices.^30^ Immune checkpoint inhibitors elicit durable and effective responses through tumor volume reduction by targeting the Programmed Cell Death-1 receptor (PD-1) and PD-Ligand 1 (PD-L1).^7^^,^^31^ Cancer cells can highly express PD-L1 to evade immune surveillance and escape immune system influence, leading to poor prognosis. In mRCC patients receiving ICI therapy, GPS/mGPS is particularly important as a prognostic score because it reflects some underlying mechanisms of immunotherapy resistance.^32^, ^33^, ^34^ Elevated CRP, a core component of GPS/mGPS, may reflect IL-6–driven systemic inflammation. Gutic et al. demonstrated that IL-6 signaling promotes MDSC expansion and PD-L1 upregulation, thereby fostering an immunosuppressive tumor microenvironment that may attenuate ICI efficacy.^7^^,^^35^, ^36^, ^37^, ^38^, ^39^, ^40^ The GPS/mGPS score, composed of both CRP and albumin, provides a solid theoretical foundation for investigating the association between GPS/mGPS and survival outcomes in RCC patients receiving immunotherapy.

This study has several limitations. The number of included studies was relatively small, precluding definitive conclusions from these endpoint analyses. Sensitivity analysis was performed only for mGPS studies; due to data limitations, subgroup analysis comparing GPS versus mGPS was not feasible. Some included studies did not adjust for IMDC, which may affect assessment of the independent prognostic value of GPS. For metastatic RCC, GPS may need to be combined with IMDC for improved survival prediction. Therefore, clinical studies remain necessary to further validate the relevance of GPS and mGPS in predicting survival for individual cancer patients, and additional research incorporating other prognostic markers is warranted to enhance predictive accuracy.

## Conclusion

This meta-analysis indicates that GPS/mGPS may act as a reliable prognostic determinant in RCC patients receiving ICI therapy. Elevated GPS/mGPS is linked to poorer OS and PFS. GPS/mGPS scoring may assist clinicians in predicting treatment outcomes. Prospective validation in more diverse patient populations is needed to facilitate broader clinical application.

## Glossary

OS, Overall Survival; GPS, Glasgow Prognostic Score; RCC, Renal Cell Carcinoma; Cis, Confidence Intervals; HRs, Hazard Ratios; PFS, Progression-Free Survival; ICI, Immune Checkpoint Inhibitor; MeSH, Medical Subject Headings; NOS, Newcastle-Ottawa Scale; mGPS, modified GPS; mUC, Urothelial Carcinoma.

## Data availability statement

The datasets analyzed during the current study are available in Supplementary Material 4.

## Ethics approval and consent to participate

As this is a meta-analysis, ethical approval is not applicable.

## Authors’ contributions

All authors contributed to the study conception and design. Writing-original draft preparation: [Xiangning Zhang]; Writing-review and editing: [Xiangning Zhang]; Conceptualization: [Xiangning Zhang]; Methodology: [Ning Shi]; Formal analysis and investigation: [Yajun Xie]; Resources: [Xiaoye Li]; Supervision: [Ning Shi], and all authors commented on previous versions of the manuscript. All authors read and approved the final manuscript.

## Funding

This research did not receive any specific grant from funding agencies in the public, commercial, or not-for-profit sectors.

## Conflicts of interest

The authors declare no conflicts of interest.

## Appendix Supplementary materials

Image, application 1

## Footnotes

Supplementary material associated with this article can be found, in the online version, at doi:10.1016/j.clinsp.2026.101008.
