# Supplementary material for: Prognostic role of the Glasgow prognostic score and modified Glasgow prognostic score in patients with renal cell carcinoma undergoing immunotherapy: a meta-analysis
Source: Clinics (Sao Paulo). 2026 Jul 17;81:101008. doi: 10.1016/j.clinsp.2026.101008 (PMC13401035; doi:10.1016/j.clinsp.2026.101008)
Supplement: Supplementary file 1 [file mmc1.docx]

**CLINICS-D-25-01868**

**Supplementary Material 1.** Search strategy.

**The search strategy (Pubmed)**

| Search number | Query | Results |
| --- | --- | --- |
| 1 | (Immunotherapy[MeSH Terms]) OR (Immune checkpoint inhibitors[MeSH Terms]) | 368,796 |
| 2 | "biologic response modifier therapy"[Title/Abstract] OR "biological response modifier therapy"[Title/Abstract] OR "BRM therapy"[Title/Abstract] OR "CTLA 4 Inhibitor"[Title/Abstract] OR "CTLA 4 Inhibitors"[Title/Abstract] OR "Cytotoxic T Lymphocyte Associated Protein 4 Inhibitor"[Title/Abstract] OR "Cytotoxic T Lymphocyte Associated Protein 4 Inhibitors"[Title/Abstract] OR "Immune Checkpoint Blockade"[Title/Abstract] OR "immune checkpoint blocker"[Title/Abstract] OR "Immune Checkpoint Blockers"[Title/Abstract] OR "Immune Checkpoint Inhibition"[Title/Abstract] OR "Immune Checkpoint Inhibitor"[Title/Abstract] OR "Immune checkpoint inhibitors"[Title/Abstract] OR "immune therapy"[Title/Abstract] OR "immunogenic therapy"[Title/Abstract] OR "immunoglobulin therapy"[Title/Abstract] OR "immunological therapy"[Title/Abstract] OR "immunological treatment"[Title/Abstract] OR "immunomodulant therapy"[Title/Abstract] OR "immunomodulary therapy"[Title/Abstract] OR "immunomodulating therapy"[Title/Abstract] OR "immunomodulation therapy"[Title/Abstract] OR "immunomodulative therapy"[Title/Abstract] OR "immunomodulator therapy"[Title/Abstract] OR "immunomodulatory intervention"[Title/Abstract] OR "immunomodulatory therapy"[Title/Abstract] OR "immunomoduling therapy"[Title/Abstract] OR "immunomodurating therapy"[Title/Abstract] OR "Immunotherapies"[Title/Abstract] OR "Immunotherapy"[Title/Abstract] OR "PD 1 Inhibitor"[Title/Abstract] OR "PD 1 Inhibitors"[Title/Abstract] OR "PD 1 PD L1 Blockade"[Title/Abstract] OR "PD L1 Inhibitor"[Title/Abstract] OR "PD L1 Inhibitors"[Title/Abstract] OR "Programmed Cell Death Protein 1 Inhibitor"[Title/Abstract] OR "Programmed Cell Death Protein 1 Inhibitors"[Title/Abstract] OR "Programmed Death Ligand 1 Inhibitors"[Title/Abstract] | 196,009 |
| 3 | Carcinoma, Renal Cell[MeSH Terms] | 43,503 |
| 4 | "Adenocarcinoma Of Kidney"[Title/Abstract] OR "Adenocarcinoma Of Kidneys"[Title/Abstract] OR "adenocarcinoma of the kidney"[Title/Abstract] OR "Chromophil Renal Cell Carcinoma"[Title/Abstract] OR "Chromophobe Renal Cell Carcinoma"[Title/Abstract] OR "Clear Cell Renal Carcinoma"[Title/Abstract] OR "Clear Cell Renal Cell Carcinoma"[Title/Abstract] OR "Collecting Duct Carcinoma"[Title/Abstract] OR "Collecting Duct Carcinoma Kidney"[Title/Abstract] OR "Collecting Duct Carcinoma of the Kidney"[Title/Abstract] OR "Collecting Duct Carcinomas"[Title/Abstract] OR "Collecting Duct Carcinomas Kidney"[Title/Abstract] OR "Grawitz Tumor"[Title/Abstract] OR "Grawitz tumour"[Title/Abstract] OR "Grawitzs tumor"[Title/Abstract] OR "Grawitzs tumour"[Title/Abstract] OR "hyper nephroma"[Title/Abstract] OR "hypernephroid cancer"[Title/Abstract] OR "Hypernephroid Carcinoma"[Title/Abstract] OR "Hypernephroid Carcinomas"[Title/Abstract] OR "Hypernephroma"[Title/Abstract] OR "Hypernephromas"[Title/Abstract] OR "kidney adenocarcinoma"[Title/Abstract] OR "kidney cell adenocarcinoma"[Title/Abstract] OR "kidney cell cancer"[Title/Abstract] OR "kidney cell carcinoma"[Title/Abstract] OR "kidney hypernephroma"[Title/Abstract] OR "kidney renal cell cancer"[Title/Abstract] OR "Nephroid Carcinoma"[Title/Abstract] OR "Nephroid Carcinomas"[Title/Abstract] OR "Papillary Renal Cell Carcinoma"[Title/Abstract] OR "Renal Adenocarcinoma"[Title/Abstract] OR "Renal Adenocarcinomas"[Title/Abstract] OR "Renal Carcinoma"[Title/Abstract] OR "Renal Carcinomas"[Title/Abstract] OR "Renal Cell Adenocarcinoma"[Title/Abstract] OR "Renal Cell Adenocarcinomas"[Title/Abstract] OR "Renal Cell Cancer"[Title/Abstract] OR "Renal Cell Cancers"[Title/Abstract] OR "Renal Cell Carcinoma"[Title/Abstract] OR "Renal Cell Carcinomas"[Title/Abstract] OR "Renal Collecting Duct Carcinoma"[Title/Abstract] OR "Sarcomatoid Renal Cell Carcinoma"[Title/Abstract] | 60,530 |
| 5 | (Glasgow Prognostic Score[Title/Abstract]) OR (GPS[Title/Abstract]) | 35,874 |
| 6 | (#1 OR #2) AND (#3 OR #4) AND #5 | 11 |

**The search strategy (embase)**

| Search number | Query | Results |
| --- | --- | --- |
| 1 | 'renal cell carcinoma'/exp | 47108 |
| 2 | 'adenocarcinoma of kidney':ab,ti,kw OR 'adenocarcinoma of kidneys':ab,ti,kw OR 'adenocarcinoma of the kidney':ab,ti,kw OR 'chromophil renal cell carcinoma':ab,ti,kw OR 'chromophobe renal cell carcinoma':ab,ti,kw OR 'clear cell renal carcinoma':ab,ti,kw OR 'clear cell renal cell carcinoma':ab,ti,kw OR 'collecting duct carcinoma':ab,ti,kw OR 'collecting duct carcinoma kidney':ab,ti,kw OR 'collecting duct carcinoma of the kidney':ab,ti,kw OR 'collecting duct carcinomas':ab,ti,kw OR 'collecting duct carcinomas kidney':ab,ti,kw OR 'grawitz tumor':ab,ti,kw OR 'grawitz tumour':ab,ti,kw OR 'grawitzs tumor':ab,ti,kw OR 'grawitzs tumour':ab,ti,kw OR 'hyper nephroma':ab,ti,kw OR 'hypernephroid cancer':ab,ti,kw OR 'hypernephroid carcinoma':ab,ti,kw OR 'hypernephroid carcinomas':ab,ti,kw OR 'hypernephroma':ab,ti,kw OR 'hypernephromas':ab,ti,kw OR 'kidney adenocarcinoma':ab,ti,kw OR 'kidney cell adenocarcinoma':ab,ti,kw OR 'kidney cell cancer':ab,ti,kw OR 'kidney cell carcinoma':ab,ti,kw OR 'kidney hypernephroma':ab,ti,kw OR 'kidney renal cell cancer':ab,ti,kw OR 'nephroid carcinoma':ab,ti,kw OR 'nephroid carcinomas':ab,ti,kw OR 'papillary renal cell carcinoma':ab,ti,kw OR 'renal adenocarcinoma':ab,ti,kw OR 'renal adenocarcinomas':ab,ti,kw OR 'renal carcinoma':ab,ti,kw OR 'renal carcinomas':ab,ti,kw OR 'renal cell adenocarcinoma':ab,ti,kw OR 'renal cell adenocarcinomas':ab,ti,kw OR 'renal cell cancer':ab,ti,kw OR 'renal cell cancers':ab,ti,kw OR 'renal cell carcinoma':ab,ti,kw OR 'renal cell carcinomas':ab,ti,kw OR 'renal collecting duct carcinoma':ab,ti,kw OR 'sarcomatoid renal cell carcinoma':ab,ti,kw | 89527 |
| 3 | 'immunotherapy'/exp OR 'immune checkpoint inhibitor'/exp | 407831 |
| 4 | 'biologic response modifier therapy':ab,ti,kw OR 'biological response modifier therapy':ab,ti,kw OR 'brm therapy':ab,ti,kw OR 'ctla 4 inhibitor':ab,ti,kw OR 'ctla 4 inhibitors':ab,ti,kw OR 'cytotoxic t lymphocyte associated protein 4 inhibitor':ab,ti,kw OR 'cytotoxic t lymphocyte associated protein 4 inhibitors':ab,ti,kw OR 'immune checkpoint blockade':ab,ti,kw OR 'immune checkpoint blocker':ab,ti,kw OR 'immune checkpoint blockers':ab,ti,kw OR 'immune checkpoint inhibition':ab,ti,kw OR 'immune checkpoint inhibitor':ab,ti,kw OR 'immune checkpoint inhibitors':ab,ti,kw OR 'immune therapy':ab,ti,kw OR 'immunogenic therapy':ab,ti,kw OR 'immunoglobulin therapy':ab,ti,kw OR 'immunological therapy':ab,ti,kw OR 'immunological treatment':ab,ti,kw OR 'immunomodulant therapy':ab,ti,kw OR 'immunomodulary therapy':ab,ti,kw OR 'immunomodulating therapy':ab,ti,kw OR 'immunomodulation therapy':ab,ti,kw OR 'immunomodulative therapy':ab,ti,kw OR 'immunomodulator therapy':ab,ti,kw OR 'immunomodulatory intervention':ab,ti,kw OR 'immunomodulatory therapy':ab,ti,kw OR 'immunomoduling therapy':ab,ti,kw OR 'immunomodurating therapy':ab,ti,kw OR 'immunotherapies':ab,ti,kw OR 'immunotherapy':ab,ti,kw OR 'pd 1 inhibitor':ab,ti,kw OR 'pd 1 inhibitors':ab,ti,kw OR 'pd 1 pd l1 blockade':ab,ti,kw OR 'pd l1 inhibitor':ab,ti,kw OR 'pd l1 inhibitors':ab,ti,kw OR 'programmed cell death protein 1 inhibitor':ab,ti,kw OR 'programmed cell death protein 1 inhibitors':ab,ti,kw OR 'programmed death ligand 1 inhibitors':ab,ti,kw | 303143 |
| 5 | glasgow AND prognostic AND score OR gps | 58019 |
| 6 | (#1 OR #2) AND (#3 OR #4) AND #5 | 26 |

**The search strategy (Cochrane)**

| Search number | Query | Results |
| --- | --- | --- |
| 1 | MeSH descriptor: [Immunotherapy] explode all trees | 12045 |
| 2 | MeSH descriptor: [Immune Checkpoint Inhibitors] explode all trees | 343 |
| 3 | (‘biologic response modifier therapy’ OR ‘biological response modifier therapy’ OR ‘BRM therapy’ OR ‘CTLA 4 Inhibitor’ OR ‘CTLA 4 Inhibitors’ OR ‘Cytotoxic T Lymphocyte Associated Protein 4 Inhibitor’ OR ‘Cytotoxic T Lymphocyte Associated Protein 4 Inhibitors’ OR ‘Immune Checkpoint Blockade’ OR ‘immune checkpoint blocker’ OR ‘Immune Checkpoint Blockers’ OR ‘Immune Checkpoint Inhibition’ OR ‘Immune Checkpoint Inhibitor’ OR ‘Immune checkpoint inhibitors’ OR ‘immune therapy’ OR ‘immunogenic therapy’ OR ‘immunoglobulin therapy’ OR ‘immunological therapy’ OR ‘immunological treatment’ OR ‘immunomodulant therapy’ OR ‘immunomodulary therapy’ OR ‘immunomodulating therapy’ OR ‘immunomodulation therapy’ OR ‘immunomodulative therapy’ OR ‘immunomodulator therapy’ OR ‘immunomodulatory intervention’ OR ‘immunomodulatory therapy’ OR ‘immunomoduling therapy’ OR ‘immunomodurating therapy’ OR ‘Immunotherapies’ OR ‘Immunotherapy’ OR ‘PD 1 Inhibitor’ OR ‘PD 1 Inhibitors’ OR ‘PD 1 PD L1 Blockade’ OR ‘PD L1 Inhibitor’ OR ‘PD L1 Inhibitors’ OR ‘Programmed Cell Death Protein 1 Inhibitor’ OR ‘Programmed Cell Death Protein 1 Inhibitors’ OR ‘Programmed Death Ligand 1 Inhibitors’):ab,ti,kw | 55980 |
| 4 | MeSH descriptor: [Carcinoma, Renal Cell] explode all trees | 1562 |
| 5 | (‘Adenocarcinoma Of Kidney’ OR ‘Adenocarcinoma Of Kidneys’ OR ‘adenocarcinoma of the kidney’ OR ‘Chromophil Renal Cell Carcinoma’ OR ‘Chromophobe Renal Cell Carcinoma’ OR ‘Clear Cell Renal Carcinoma’ OR ‘Clear Cell Renal Cell Carcinoma’ OR ‘Collecting Duct Carcinoma’ OR ‘Collecting Duct Carcinoma Kidney’ OR ‘Collecting Duct Carcinoma of the Kidney’ OR ‘Collecting Duct Carcinomas’ OR ‘Collecting Duct Carcinomas Kidney’ OR ‘Grawitz Tumor’ OR ‘Grawitz tumour’ OR ‘Grawitzs tumor’ OR ‘Grawitzs tumour’ OR ‘hyper nephroma’ OR ‘hypernephroid cancer’ OR ‘Hypernephroid Carcinoma’ OR ‘Hypernephroid Carcinomas’ OR ‘Hypernephroma’ OR ‘Hypernephromas’ OR ‘kidney adenocarcinoma’ OR ‘kidney cell adenocarcinoma’ OR ‘kidney cell cancer’ OR ‘kidney cell carcinoma’ OR ‘kidney hypernephroma’ OR ‘kidney renal cell cancer’ OR ‘Nephroid Carcinoma’ OR ‘Nephroid Carcinomas’ OR ‘Papillary Renal Cell Carcinoma’ OR ‘Renal Adenocarcinoma’ OR ‘Renal Adenocarcinomas’ OR ‘Renal Carcinoma’ OR ‘Renal Carcinomas’ OR ‘Renal Cell Adenocarcinoma’ OR ‘Renal Cell Adenocarcinomas’ OR ‘Renal Cell Cancer’ OR ‘Renal Cell Cancers’ OR ‘Renal Cell Carcinoma’ OR ‘Renal Cell Carcinomas’ OR ‘Renal Collecting Duct Carcinoma’ OR ‘Sarcomatoid Renal Cell Carcinoma’):ab,ti,kw | 8328 |
| 6  7 | Glasgow Prognostic Score OR gps  (#1 OR #2 OR #3) AND (#4 OR #5) AND #6 | 4663  3 |

**The search strategy (Web of Science)**

| Search number | Query | Results |
| --- | --- | --- |
| 1 | "TS=((biologic response modifier therapy) OR (biological response modifier therapy) OR (BRM therapy) OR (CTLA 4 Inhibitor) OR (CTLA 4 Inhibitors) OR (Cytotoxic T Lymphocyte Associated Protein 4 Inhibitor) OR (Cytotoxic T Lymphocyte Associated Protein 4 Inhibitors) OR (Immune Checkpoint Blockade) OR (immune checkpoint blocker) OR (Immune Checkpoint Blockers) OR (Immune Checkpoint Inhibition) OR (Immune Checkpoint Inhibitor) OR (Immune checkpoint inhibitors) OR (immune therapy) OR (immunogenic therapy) OR (immunoglobulin therapy) OR (immunological therapy) OR (immunological treatment) OR (immunomodulant therapy) OR (immunomodulary therapy) OR (immunomodulating therapy) OR (immunomodulation therapy) OR (immunomodulative therapy) OR (immunomodulator therapy) OR (immunomodulatory intervention) OR (immunomodulatory therapy) OR (immunomoduling therapy) OR (immunomodurating therapy) OR (Immunotherapies) OR (Immunotherapy) OR (PD 1 Inhibitor) OR (PD 1 Inhibitors) OR (PD 1 PD L1 Blockade) OR (PD L1 Inhibitor) OR (PD L1 Inhibitors) OR (Programmed Cell Death Protein 1 Inhibitor) OR (Programmed Cell Death Protein 1 Inhibitors) OR (Programmed Death Ligand 1 Inhibitors)) and Preprint Citation Index (Exclude – Database) " | 2106302 |
| 2 | "TS=((Adenocarcinoma Of Kidney) OR (Adenocarcinoma Of Kidneys) OR (adenocarcinoma of the kidney) OR (Chromophil Renal Cell Carcinoma) OR (Chromophobe Renal Cell Carcinoma) OR (Clear Cell Renal Carcinoma) OR (Clear Cell Renal Cell Carcinoma) OR (Collecting Duct Carcinoma) OR (Collecting Duct Carcinoma Kidney) OR (Collecting Duct Carcinoma of the Kidney) OR (Collecting Duct Carcinomas) OR (Collecting Duct Carcinomas Kidney) OR (Grawitz Tumor) OR (Grawitz tumour) OR (Grawitzs tumor) OR (Grawitzs tumour) OR (hyper nephroma) OR (hypernephroid cancer) OR (Hypernephroid Carcinoma) OR (Hypernephroid Carcinomas) OR (Hypernephroma) OR (Hypernephromas) OR (kidney adenocarcinoma) OR (kidney cell adenocarcinoma) OR (kidney cell cancer) OR (kidney cell carcinoma) OR (kidney hypernephroma) OR (kidney renal cell cancer) OR (Nephroid Carcinoma) OR (Nephroid Carcinomas) OR (Papillary Renal Cell Carcinoma) OR (Renal Adenocarcinoma) OR (Renal Adenocarcinomas) OR (Renal Carcinoma) OR (Renal Carcinomas) OR (Renal Cell Adenocarcinoma) OR (Renal Cell Adenocarcinomas) OR (Renal Cell Cancer) OR (Renal Cell Cancers) OR (Renal Cell Carcinoma) OR (Renal Cell Carcinomas) OR (Renal Collecting Duct Carcinoma) OR (Sarcomatoid Renal Cell Carcinoma)) and Preprint Citation Index (Exclude – Database) " | 245764 |
| 3 | "TS=(Glasgow Prognostic Score OR GPS) and Preprint Citation Index (Exclude – Database) " | 105704 |
| 4 | "#3 AND #2 AND #1 and Preprint Citation Index (Exclude – Database) " | 38 |

**Supplementary Material 2.** Information of 9 articles included

1. Brown JT, Liu Y, Shabto JM, Martini D, Ravindranathan D, Hitron EE et al. Modified Glasgow Prognostic Score associated with survival in metastatic renal cell carcinoma treated with immune checkpoint inhibitors. J Immunother Cancer. 2021;9(7)

2. Fujiwara R, Takemura K, Fujiwara M, Yuasa T, Yasuoka S, Komai Y et al. Modified Glasgow Prognostic Score as a Predictor of Prognosis in Metastatic Renal Cell Carcinoma Treated With Nivolumab. Clin Genitourin Cancer. 2021;19(2):e78-e83.

3. Minichsdorfer C, Gleiss A, Aretin MB, Schmidinger M, Fuereder T. Serum parameters as prognostic biomarkers in a real world cancer patient population treated with anti PD-1/PD-L1 therapy. Ann Med. 2022;54(1):1339-1349.

4. Noguchi G, Nakaigawa N, Umemoto S, Kobayashi K, Shibata Y, Tsutsumi S et al. C-reactive protein at 1 month after treatment of nivolumab as a predictive marker of efficacy in advanced renal cell carcinoma. Cancer Chemother Pharmacol. 2020;86(1):75-85.

5. Saal J, Bald T, Eckstein M, Ralser DJ, Ritter M, Brossart P et al. Integrating On-Treatment Modified Glasgow Prognostic Score and Imaging to Predict Response and Outcomes in Metastatic Renal Cell Carcinoma. JAMA Oncol. 2023;9(8):1048-1055.

6. Saal J, Eckstein M, Ritter M, Brossart P, Hölzel M, Grünwald V et al. The modified Glasgow Prognostic Score (mGPS) can guide decisions for immunotherapy treatment beyond progression. Eur J Cancer. 2025;215:115163.

7. Sato MT, Ida A, Kanda Y, Takano K, Ohbayashi M, Kohyama N et al. Prognostic model for overall survival that includes the combination of platelet count and neutrophil-lymphocyte ratio within the first six weeks of sunitinib treatment for metastatic renal cell carcinoma. BMC Cancer. 2022;22(1):1214.

8. Walach MT, Burger R, Brumm F, Nitschke K, Wessels F, Nuhn P et al. Prognostic scores for predicting overall survival in patients with metastatic renal and urothelial cancer undergoing immunotherapy - which one to use? World J Urol. 2025;43(1):93.

9. Yildirim A, Wei M, Liu Y, Nazha B, Brown JT, Carthon BC et al. Association of baseline inflammatory biomarkers and clinical outcomes in patients with advanced renal cell carcinoma treated with immune checkpoint inhibitors. Ther Adv Med Oncol. 2025;17:17588359251316243.

**Supplementary Material 3.** Quality assessment

**NEWCASTLE - OTTAWA QUALITY ASSESSMENT SCALE**

**COHORT STUDIES**

Note: A study can be awarded a maximum of one star for each numbered item within the Selection and Outcome categories. A maximum of two stars can be given for Comparability

**Selection**

1) Representativeness of the exposed cohort

a) truly representative of the average _______________ (describe) in the community **🟑**

b) somewhat representative of the average ______________ in the community **🟑**

c) selected group of users eg nurses, volunteers

d) no description of the derivation of the cohort

2) Selection of the non exposed cohort

a) drawn from the same community as the exposed cohort **🟑**

b) drawn from a different source

c) no description of the derivation of the non exposed cohort

3) Ascertainment of exposure

a) secure record (eg surgical records) **🟑**

b) structured interview **🟑**

c) written self report

d) no description

4) Demonstration that outcome of interest was not present at start of study

a) yes **🟑**

b) no

**Comparability**

1) Comparability of cohorts on the basis of the design or analysis

a) study controls for _____________ (select the most important factor) **🟑**

b) study controls for any additional factor **🟑** (This criteria could be modified to indicate specific control for a second important factor.)

**Outcome**

1) Assessment of outcome

a) independent blind assessment **🟑**

b) record linkage **🟑**

c) self report

d) no description

2) Was follow-up long enough for outcomes to occur

a) yes (select an adequate follow up period for outcome of interest) **🟑**

b) no

3) Adequacy of follow up of cohorts

a) complete follow up - all subjects accounted for **🟑**

b) subjects lost to follow up unlikely to introduce bias - small number lost - > ____ % (select an adequate %) follow up, or description provided of those lost) **🟑**

c) follow up rate < ____% (select an adequate %) and no description of those lost

d) no statement

| Author, year | selection | | | | Comparability | Outcome | | | Total Score |
| --- | --- | --- | --- | --- | --- | --- | --- | --- | --- |
|  | #1 | #2 | #3 | #4 | #1 | #1 | #2 | #3 |  |
| Brown, J. T.2021 | 1 | 1 | 1 | 0 | 2 | 1 | 1 | 1 | 8 |
| Fujiwara, R.2020 | 1 | 1 | 1 | 0 | 2 | 1 | 0 | 1 | 7 |
| Minichsdorfer, C.2022 | 1 | 1 | 1 | 0 | 1 | 1 | 1 | 1 | 7 |
| Saal, J.2025 | 1 | 1 | 1 | 0 | 1 | 1 | 1 | 1 | 7 |
| Sato, M. T.2022 | 1 | 1 | 1 | 0 | 2 | 1 | 1 | 1 | 8 |
| Walach, M. T.2025 | 1 | 1 | 1 | 0 | 2 | 1 | 1 | 1 | 8 |
| Yildirim, A.2025 | 1 | 1 | 1 | 0 | 2 | 1 | 1 | 1 | 8 |
| Noguchi, Go2020 | 1 | 1 | 1 | 0 | 2 | 1 | 1 | 1 | 8 |
| Saal, Jonas2023 | 1 | 1 | 1 | 0 | 2 | 1 | 1 | 1 | 8 |
| Tran, Steven D.2025 | 1 | 1 | 1 | 0 | 1 | 1 | 1 | 1 | 7 |

**Supplementary Material 4** Original data for overall survival and progression-free survival.

1. **Original data for overall survival (OS)**

| **Author** | **Year** | HR | **lci** | **uci** | **metastasis** | **number** | **area** | **drug** | **Categories** | **Semple size** |
| --- | --- | --- | --- | --- | --- | --- | --- | --- | --- | --- |
| Brown, J. T. | 2021 | 2.508 | 1.547 | 4.068 | Metastasis | >100 | North America | Combination | mGPS | 156 |
| Fujiwara, R. | 2020 | 4.29 | 1 | 18.37 | Metastasis | <100 | Asia | Monotherapy | mGPS | 45 |
| Minichsdorfer, C. | 2022 | 2.167 | 1.045 | 4.492 | Non-metastasis | >100 | Europe | Combination | GPS | 114 |
| Saal, J. | 2025 | 2.184 | 1.335 | 3.572 | Non-metastasis | >100 | Europe | Combination | mGPS | 121 |
| Sato, M. T. | 2022 | 2.946 | 1.3855 | 6.2642 | Non-metastasis | >100 | Asia | Monotherapy | mGPS | 102 |
| Walach, M. T. | 2025 | 3.219 | 1.841 | 5.627 | Metastasis | <100 | Europe | Combination | mGPS | 53 |
| Yildirim, A. | 2025 | 1.548 | 1.046 | 2.294 | Non-metastasis | >100 | North America | Combination | mGPS | 401 |
| Saal, Jonas | 2023 | 3.629 | 2.428 | 5.425 | Metastasis | >100 | Europe | Combination | mGPS | 691 |

**B, Supplementary Material 3B. Original data for progression-free survival (PFS).**

| **Author** | **Year** | **HR** | **lci** | **uci** | **number** | **Area** |
| --- | --- | --- | --- | --- | --- | --- |
| Brown, J. T. | 2021 | 1.471 | 1.069 | 2.025 | >100 | America |
| Saal, J. | 2025 | 1.909 | 1.211 | 3.008 | >100 | Europe |
| Sato, M. T. | 2022 | 2.134 | 1.3587 | 3.3518 | >100 | Asia |
| Yildirim, A. | 2025 | 1.876 | 1.432 | 2.457 | >100 | America |
| Noguchi, Go | 2020 | 2.88 | 1.13 | 7.34 | <100 | Asia |
| Saal, Jonas | 2023 | 1.901 | 1.509 | 2.396 | >100 | America |
